# Coexistent Brugada Syndrome and Wolff-Parkinson-White Syndrome: What is the Optimal Management?

**DOI:** 10.1016/s0972-6292(16)30669-6

**Published:** 2013-09-01

**Authors:** Abhishek Jaiswal, Katherine Heretis, Seth Goldbarg

**Affiliations:** New York Hospital Queens/ Weill Cornell Medical College, Flushing, New York-11365, USA.

**Keywords:** Brugada syndrome, WPW syndrome, accessory pathway, sudden cardiac death, implantable defibrillator

## Abstract

Coexistent Brugada syndrome and Wolff-Parkinson-White (WPW) syndrome is rare, and as such poses management challenges. The overlap of symptoms attributable to each condition, the timing of ventricular stimulation after accessory pathway ablation and the predictive value of programmed stimulation in Brugada syndrome are controversial. We describe a case of coexistent Brugada syndrome and WPW syndrome in a symptomatic young adult. We discuss our treatment approach and the existing literature along with the challenges in management of such cases.

## Introduction

Both cardiac channelopathies and ventricular pre-excitation are important causes of sudden cardiac death in young adults. The coexistence of these conditions appears to be rare, and as such poses management challenges. Advances in translational medicine between molecular genetics, experimental, and clinical electrophysiology have begun to shed light on the genetic background of primary electrical disorders. However, the relationship between genetic mutations and clinical conditions are both complex and only beginning to be understood. We describe a case of coexistent Brugada syndrome and Wolff-Parkinson-White (WPW) syndrome in a symptomatic young adult.

## Case Report

**Figure 1:**
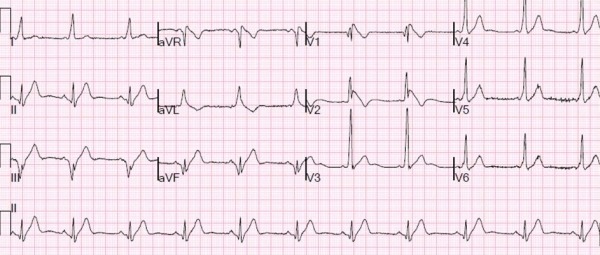
Initial electrocardiogram (ECG) showed normal sinus rhythm with evidence of posteroseptal pre-excitation, incomplete right bundle branch block, and ST segment elevations in V1-V2.

A 23-year-old otherwise healthy Hispanic male presented to the emergency department with one month of paroxysmal palpitations associated with lightheadedness and presyncope. The patient denied any prior true syncopal episode or any history of sudden cardiac death in his family. An electrocardiogram (ECG) showed normal sinus rhythm with evidence of right septal pre-excitation, incomplete right bundle branch block, and ST segment elevation in V1-V2 consistent with type 1 Brugada pattern (Figure 1). Physical examination and serum electrolytes were within the normal range. A chest X-ray was unremarkable.

**Figure 2:**
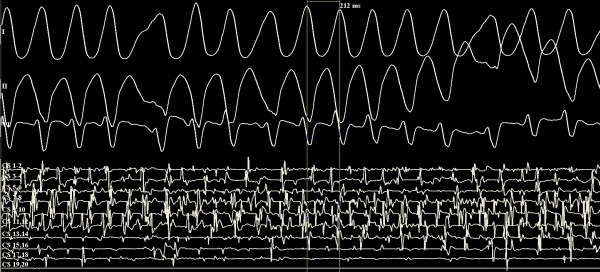
Pre-excited atrial fibrillation was induced during catheter manipulation. The shortest RR interval was 210 milliseconds.

**Figure 3:**
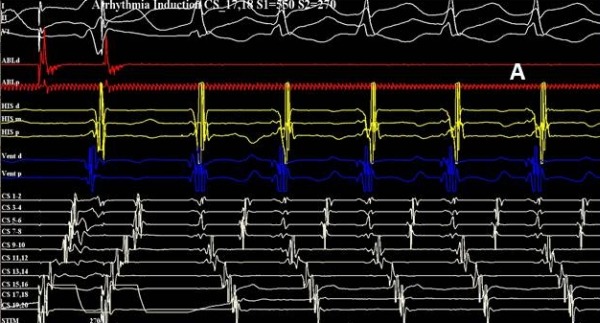

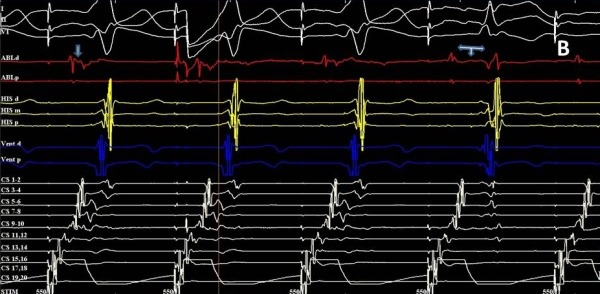
A) Orthodromic AVRT was induced during atrial extrastimulus testing. B) The successful right posteroseptal site shows fused atrial and ventricular signals during atrial pacing (simple arrow); termination of pathway conduction occurred with < 1 second RF energy, leading to widely split atrial and ventricular signals (split arrow). ABL- ablation catheter. HIS- His catheter, Vent-Ventricular catheter, CS Orbiter catheter with 2 millimeter interelectrode spacing. CS Orbiter 19-20: High RA; CS Orbiter 7-8: CS Os; CS Orbiter 1-2: Distal CS recordings.

Based on the ECG findings and the recurrent symptoms the patient underwent electrophysiological study. Endocardial electrograms were bandpass filtered at 30-300 Hz and recorded using a computerized multichannel EP workmate 4.1 (St. Jude Medical, Inc, USA). Baseline conduction intervals were notable for a short HV interval of 16 milliseconds (msec). The AH interval was 60 msec, QRS duration 118 msec, and RR interval 1067 msec. Diagnostic catheters were placed via femoral access in the high right atrium (CRD, St. Jude Medical), His region (CRD-2, St. Jude Medical), and RV apex (CRD, St. Jude Medical), as well as the coronary sinus (Orbiter, Bard Electrophysiology, USA). During catheter manipulation, pre-excited atrial fibrillation was induced with the shortest RR interval of 210 milliseconds (Figure 2). The patient became agitated and the blood pressure was unobtainable, and the patient was successfully cardioverted with a single 50 Joule biphasic shock. Atrial extrastimulus testing then induced sustained orthodromic AV reentrant tachycardia (AVRT) with a cycle length of 370 msec which was terminated with overdrive pacing (Figure 3A). A 7-French steerable, quadripolar, deflectable ablation catheter with a 4 mm distal electrode (Biosense Webster Inc, Celsius 35E-37E, Baldwin Park, CA, U.S.A.) was introduced into the right atrium. Mapping using the Ensite NavXTM (St. Jude Medical) 3-dimensional system was carried out during atrial pacing to maximize preexcitation. The earliest ventricular activation was localized to the right posterior septum (Figure 3B). Radiofrequency (RF) energy was delivered using a conventional generator (Biosense Webster Stockert 70 RF), with continuous monitoring of power, impedance and temperature. At the successful site, preexcitation terminated after 0.8 seconds of 40 watt energy delivery. There was no evidence of pre-excitation after an observation interval of 30 minutes.

**Figure 4:**
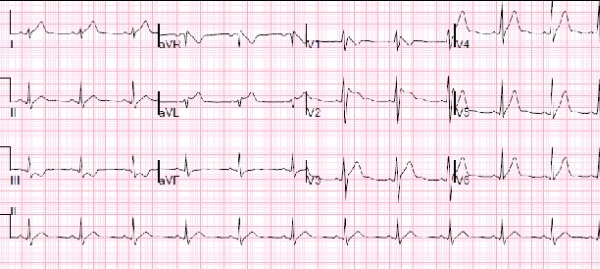
Brugada pattern on the surface electrocardiogram persisted at the conclusion of the AP ablation.

Post ablation, the type 1 Brugada pattern persisted on ECG (Figure 4). Programmed ventricular stimulation was carried out at the right ventricular (RV) apex and RV outflow tract at two different baseline drive train cycle lengths of 520 msec and 450 msec with up to triple extrastimuli. During ventricular stimulation of the outflow tract, VF was induced at a baseline drive train cycle length of 450 msec and extra stimuli of 260 msec/ 200 msec /200 msec. An initial attempt to cardiovert at 200 joules failed. A second shock of 300 joules converted the patient to sinus rhythm.

The following day an ECG showed a type 2 Brugada pattern and no pre-excitation. An implantable cardioverter defibrillator (ICD) was implanted after discussion with the patient. However, patient refused genetic testing. The patient had no siblings or children.

## Discussion

Brugada Syndrome is described as a clinical condition associated with characteristic ECG findings and an increased risk of sudden death due to ventricular arrhythmias [1]. Characteristic ECG findings include right bundle branch block and ST segment elevation in precordial leads V1 to V3 in the absence of electrolyte abnormalities, myocardial ischemia and structural heart disease. Most cases where a discrete genetic disorder is identified are associated with an ion channel gene mutation (SCN5A) located on chromosome 3 causing loss of function of the sodium channel [2]. This sodium channel blockade facilitates the loss of the plateau phase of the action potential. Because the right ventricular epicardium has much higher density of transient outward current (Ito), the ventricular arrhythmia associated with Brugada Syndrome is right ventricular in origin [3]. An ICD is the only proven intervention in aborting sudden death in such patients. ICD is indicated in symptomatic patients and asymptomatic patients with a Type 1 pattern who develop inducible ventricular fibrillation during electrophysiological study, although the predictive value of EP study is controversial [1,4].

The Brugada syndrome together with the WPW syndrome has been reported before in the medical literature [5,6]. However, in all the described cases the patients had either Brugada syndrome or WPW syndrome initially, and during electrophysiological study or subsequent follow up developed evidence of the other condition. To the best of our knowledge, this is the first case of simultaneous Brugada syndrome and WPW syndrome reported in the literature. We thought that the repolarization pattern in our patient might possibly have resulted from abnormal repolarization associated with AP conduction, but it remained unchanged following AP ablation. Reported cases have all been described in young adults. This might be explained due to the possibly relative high mortality of such patients who do not come to diagnosis, or to the tendency of some accessory pathways to lose their conduction function over time [7]. Post-mortem studies of asymptomatic patients with WPW pattern ECG have found that in 10% of cases a cause of death cannot be determined [7]. One might speculate that while some of these patients might have been predisposed to ventricular fibrillation due to a rapidly conducting antegrade accessory pathway, other patients might have had unrecognized channelopathies. However, at this time there is no data to suggest a formal relationship between these two entities, and the WPW syndrome and the Brugada syndrome are regarded as two separate diseases.

The optimal approach for sudden death prevention in these patients is unclear, and our case raises a number of interesting questions. First, our patient's symptoms might have been attributable to a tachyarrhythmia arising from either WPW or Brugada syndrome. Did our patient have just a Type 1 Brugada pattern ECG, or true Brugada syndrome? He had not had true syncope or observed nocturnal apnea, but his presyncopal symptoms were profound. Secondly, how should this patient be treated and risk stratified? EP study and ablation of a rapidly conducting accessory pathway is curative of WPW syndrome and the possibility of associated sudden death. Programmed ventricular stimulation in a Type 1 Brugada patient is reasonable, even without symptoms, but nonetheless remains controversial. Finally, it is possible that the altered repolarization of the ventricular myocardium in the period after loss of AP conduction might have affected the inducibility of ventricular fibrillation. Memory T-waves are common and may persist for weeks after ablation of manifest accessory pathways, reflecting this altered repolarization phenomenon [8]. In our patient we elected to perform ventricular stimulation shortly after AP ablation as catheters were already in place. An alternative strategy would have been to consider use of a wearable defibrillator for several weeks prior to programmed ventricular stimulation. We used a conservative approach towards minimizing sudden cardiac death in our young patient but the optimal strategy is uncertain.
